# Functional Wnt Signaling Is Increased in Idiopathic Pulmonary Fibrosis

**DOI:** 10.1371/journal.pone.0002142

**Published:** 2008-05-14

**Authors:** Melanie Königshoff, Nisha Balsara, Eva-Maria Pfaff, Monika Kramer, Izabella Chrobak, Werner Seeger, Oliver Eickelberg

**Affiliations:** Department of Medicine, University of Giessen Lung Center, University of Giessen, Giessen, Germany; Monash University, Australia

## Abstract

**Background:**

Idiopathic pulmonary fibrosis (IPF) is a fatal lung disease, characterized by distorted lung architecture and loss of respiratory function. Alveolar epithelial cell injury and hyperplasia, enhanced extracellular matrix deposition, and (myo)fibroblast activation are features of IPF. Wnt/β-catenin signaling has been shown to determine epithelial cell fate during development. As aberrant reactivation of developmental signaling pathways has been suggested to contribute to IPF pathogenesis, we hypothesized that Wnt/β-catenin signaling is activated in epithelial cells in IPF. Thus, we quantified and localized the expression and activity of the Wnt/β-catenin pathway in IPF.

**Methodology/Principal Findings:**

The expression of Wnt1, 3a, 7b, and 10b, the Wnt receptors Fzd1-4, Lrp5-6, as well as the intracellular signal transducers Gsk-3β, β-catenin, Tcf1, 3, 4, and Lef1 was analyzed in IPF and transplant donor lungs by quantitative real-time (q)RT-PCR. Wnt1, 7b and 10b, Fzd2 and 3, β-catenin, and Lef1 expression was significantly increased in IPF. Immunohistochemical analysis localized Wnt1, Wnt3a, β-catenin, and Gsk-3β expression largely to alveolar and bronchial epithelium. This was confirmed by qRT-PCR of primary alveolar epithelial type II (ATII) cells, demonstrating a significant increase of Wnt signaling in ATII cells derived from IPF patients. In addition, Western blot analysis of phospho-Gsk-3β, phospho-Lrp6, and β-catenin, and qRT-PCR of the Wnt target genes cyclin D1, Mmp 7, or Fibronectin 1 demonstrated increased functional Wnt/β-catenin signaling in IPF compared with controls. Functional *in vitro* studies further revealed that Wnt ligands induced lung epithelial cell proliferation and (myo)fibroblast activation and collagen synthesis.

**Conclusions/Significance:**

Our study demonstrates that the Wnt/β-catenin pathway is expressed and operative in adult lung epithelium. Increased Wnt/β-catenin signaling may be involved in epithelial cell injury and hyperplasia, as well as impaired epithelial-mesenchymal cross-talk in IPF. Thus, modification of Wnt signaling may represent a therapeutic option in IPF.

## Introduction

Pulmonary fibrosis can result from a variety of causes, including lung injury, environmental particle and toxin inhalation, chemotherapy, systemic autoimmune diseases, or as an idiopathic entity in form of idiopathic interstitial pneumonias (IIP) [1]–[4]. Idiopathic pulmonary fibrosis (IPF), the most common form of IIP, represents a progressive and lethal disorder with unresolved pathogenesis and unresponsiveness to currently available therapies [5]. Distortion of the normal lung architecture in IPF is evident by temporo-spatially heterogeneous histology, including areas of normal parenchyma, mild interstitial inflammation due to mononuclear infiltrates, septal fibrosis with subepithelial fibroblast foci, and honeycombing [6], [7].

Fibroblast foci represent the hallmark lesions of IPF, as they constitute aggregates of activated myofibroblasts, which promote excessive ECM deposition [7]. The occurrence of fibroblast foci represents an important prognostic factor, since their numbers have been correlated with survival in IPF [8]. Fibroblast foci occur in subepithelial layers, close to areas of alveolar epithelial cell injury and repair, suggesting that impaired epithelial-mesenchymal crosstalk contributes to the pathobiology of IPF [8], [9]. Indeed, it is well accepted that repetitive injury and subsequent repair of alveolar epithelial type II (ATII) cells, in the presence or absence of local inflammation, represent a key pathogenic mechanism in IPF, which leads to aberrant growth factor activation and perpetuation of fibrotic transformation [10]. Although several soluble mediators, such as transforming growth factor (TGF)-β1 or interleukin (IL)-1β, have been assigned a clear pathogenic role in IPF and experimental models thereof (9, 10), therapeutic options neutralizing their activity have not been successful in clinical use as of yet.

The Wnt family constitutes a large family of highly conserved secreted growth factors essential to organ development, a process often recapitulated in organ failure. The best characterized Wnt signaling pathway is the β-catenin-dependent, or canonical, Wnt signaling pathway [11]–[13]. Here, in the absence of active Wnt ligands, β-catenin is constitutively phosphorylated by its interaction with axin, adenomatosis polyposis coli (APC), and glycogen synthase kinase (Gsk)-3β, and subsequently degraded. In the presence of Wnt ligands, two distinct membrane receptors, the frizzled (Fzd) or the low density lipoprotein receptor-related proteins (Lrp) 5 and 6, are activated upon ligand binding. In detail, Wnt stimulation leads to phosphorylation of Lrp6 by Gsk-3β and casein kinase γ in its cytoplasmic region, which leads to the recruitment of axin. Subsequently, β-catenin phosphorylation is attenuated, its degradation inhibited, and accumulated β-catenin undergoes nuclear translocation, where it regulates target gene expression through interaction with members of the T-cell-specific transcription factor/lymphoid enhancer-binding factor (Tcf/Lef) family [11], [12].

Importantly, increased nuclear β-catenin staining was recently reported in IPF tissue sections [14], indicative of increased Wnt signaling. In addition, unbiased microarray screens have also revealed an increased expression of Wnt target genes, such as matrix metalloproteinase (Mmp) 7, or secreted frizzled-related protein (Sfrp) 2 in IPF [15]–[17]. We therefore hypothesized that canonical Wnt signaling is aberrantly activated in IPF, recapitulating developmentally active programs in this chronic disease. To this end, we achieved our aim to elucidate the expression, localization, and activity of the Wnt/β-catenin pathway in IPF.

## Results

**Figure 1:**
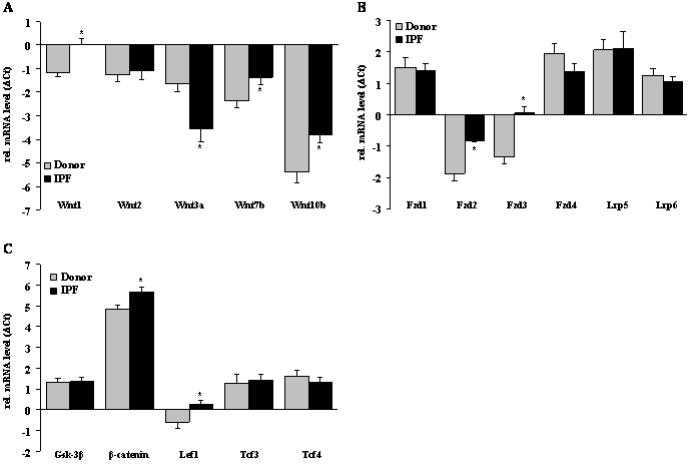
The mRNA expression profile of canonical Wnt signaling components in IPF. The mRNA levels of the Wnt ligands Wnt1, 2, 3a, 7b, and 10b (a), the receptors frizzled (Fzd) 1–4, low density lipoprotein-related protein (Lrp) 5 and 6 (b), and the intracellular signal transducers glycogen synthase kinase (Gsk)-3β, β-catenin, T-cell-specific transcription facor (Tcf) 3, Tcf 4, lymphoid enhancer-binding factor (Lef) 1 (c) were assessed in donor and IPF lung specimen by quantitative real-time PCR (qRT-PCR). Results are derived from 12 donors and 12 IPF patients and presented as mean±s.e.m., * p<0.05.

Initially, we sought to quantify the mRNA expression of canonical Wnt/β-catenin signaling components in lung tissue samples of transplant donors and IPF patients using quantitative real-time (q)RT-PCR. As depicted in Figure 1a, canonical Wnt ligands were variably expressed in the human lung. Wnt1, 2, 3a, and 7b were expressed at similar levels in normal lung tissue, while Wnt10b was only little expressed. In IPF lung specimens, Wnt1, 7b, and 10b mRNA levels were markedly upregulated (log-fold change of 1.19±0.43, 1.05±0.43, and 1.58±0.59, respectively), whereas Wnt3a was significantly downregulated (log-fold change −1.93±0.65) (Figure 1a).

Next, we analyzed the expression of common Wnt receptors and co-receptors. As shown in Figure 1b, the most abundant receptors in the human lung were Fzd1 and 4, and the co-receptors Lrp5 and 6, but their expression was similar in control and IPF lungs. Interestingly, Fzd2 and 3 were expressed at low levels in control as well as IPF lungs, but significantly increased in IPF (log-fold change 1.04±0.24 and 1.41±0.31, respectively) (Figure 1b).

The main canonical Wnt signal transducers Gsk-3β and β-catenin were both expressed in normal and fibrotic lung tissue, with a significantly increased expression of β-catenin in IPF (log-fold change 0.98±0.28) (Figure 1c). With the exception of Tcf1, all members of the Tcf/Lef family of transcription factors were expressed in normal and fibrotic lung tissue. Lef1 was significantly upregulated in IPF (log-fold change 0.85±0.34) (Figure 1c).

**Figure 2:**
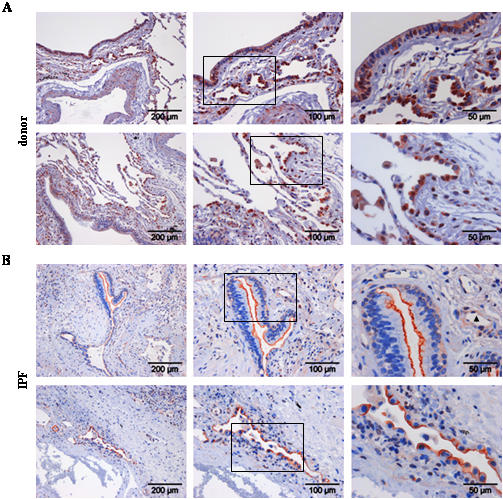
Expression and localization of Wnt1 in lung tissues of donor and IPF patients. Immunohistochemical staining was performed on tissue sections of donor (a) or IPF lungs (b). Representative pictures with focus on the bronchial (upper panel) or alveolar epithelium (lower panel) are given. Stainings are representative of two independent experiments using at least three different donor or IPF lung tissues (magnification as indicated). Arrowhead indicates positive endothelial cells.

**Figure 3:**
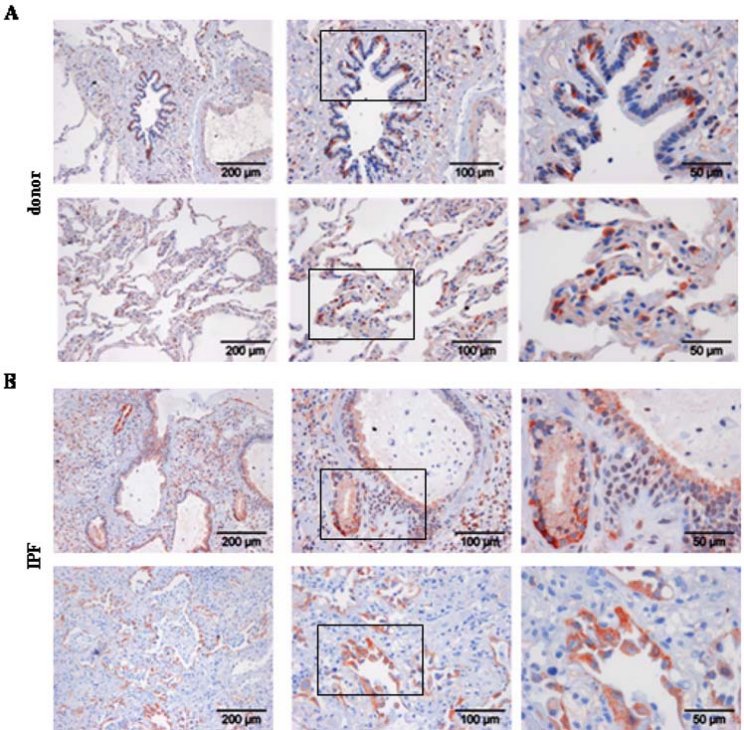
Expression and localization of Wnt3a in lung tissues of donor and IPF patients. Immunohistochemical staining was performed on tissue sections of donor (a) or IPF lungs (b). Representative pictures with focus on the bronchial (upper panel) or alveolar epithelium (lower panel) are given. Stainings are representative of two independent experiments using at least three different donor or IPF lung tissues (magnification as indicated).

**Figure 4:**
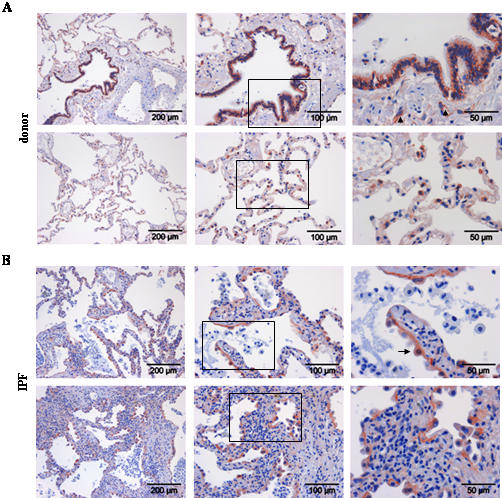
Expression and localization of total β-catenin in lung tissues of donor and IPF patients. Immunohistochemical staining was performed on tissue sections of donor (a) or IPF lungs (b). Representative pictures with focus on the bronchial (upper panel) or alveolar epithelium (lower panel) are given. Stainings are representative of two independent experiments using at least three different donor or IPF lung tissues (magnification as indicated). Arrow indicates nuclear staining of β-catenin. Arrowhead indicates positive endothelial cells.

**Figure 5:**
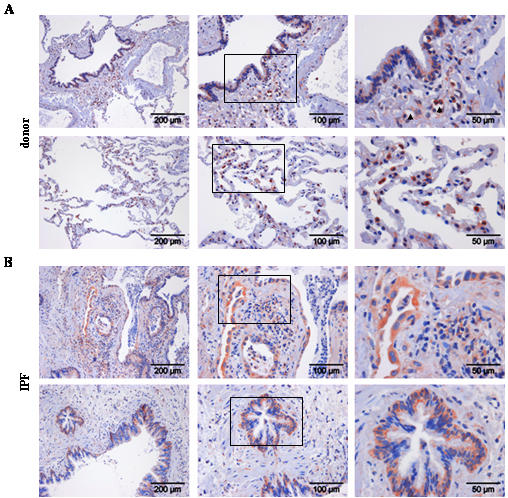
Expression and localization of total Gsk-3β in lung tissues of donor and IPF patients. Immunohistochemical staining was performed on tissue sections of donor (a) or IPF lungs (b). Representative pictures with focus on the bronchial (upper panel) or alveolar epithelium (lower panel) are given. Stainings are representative of two independent experiments using at least three different donor or IPF lung tissues (magnification as indicated). Arrowhead indicates positive endothelial cells.

We went on to localize cell types capable of Wnt ligand secretion, as assessed by immunohistochemistry of Wnt1 and 3a ligands, and cell types capable of Wnt signaling, as assessed by immunohistochemistry of β-catenin and Gsk-3β, in donor and IPF lung tissue (Figure 2, 3, 4, 5). Wnt1 was mainly expressed in bronchial and alveolar epithelium, with strong staining of alveolar epithelial type II (ATII) cells (Figure 2). Additionally, Wnt1 was expressed in vascular smooth muscle cells (Figure 2a, upper panel). In IPF (Figure 2b), Wnt1 staining was observed in hyperplastic ATII cells and bronchial epithelial cells. An apical staining pattern of Wnt1 in bronchial epithelial cells was observed in IPF, suggesting increased secretion of Wnt1. Interestingly, Wnt1 was also expressed by endothelial cells in IPF tissues (Figure 2b, lower panel, arrowhead). Wnt3a protein expression was mainly detected in ATII cells (Figure 3a, b, lower panels) and selected ciliated bronchial epithelial cells (Figure 3a, b, upper panels) in donor as well as IPF lung tissue.

We then analyzed protein expression pattern of β-catenin and Gsk-3β (Figure 4 and 5, respectively). Strong membranous and cytoplasmic β-catenin expression was observed in bronchial epithelial and ATII cells, as well as endothelial cells in donor lung tissue (Figure 4a, arrowhead). Importantly, in IPF, β-catenin expression was less membranous and enhanced in the cytoplasm, with clear nuclear staining observed in single cells (Figure 4b, arrow). Strong β-catenin expression was noticed in hyperplastic ATII cells, in particular in areas of bronchiolization (Figure 4b), as assessed by immunohistochemistry. Gsk-3β showed a very similar expression pattern to β-catenin, with predominant staining in bronchial epithelial and ATII cells, as well as endothelial cells in donor lung tissues (Figure 5a, arrowhead). In IPF lungs, intense staining of basal bronchial epithelial and hyperplastic ATII cells was observed (Figure 5b). Taken together, the Wnt/β-catenin system was largely expressed in the bronchial and alveolar epithelium in normal and IPF tissue.

**Figure 6:**
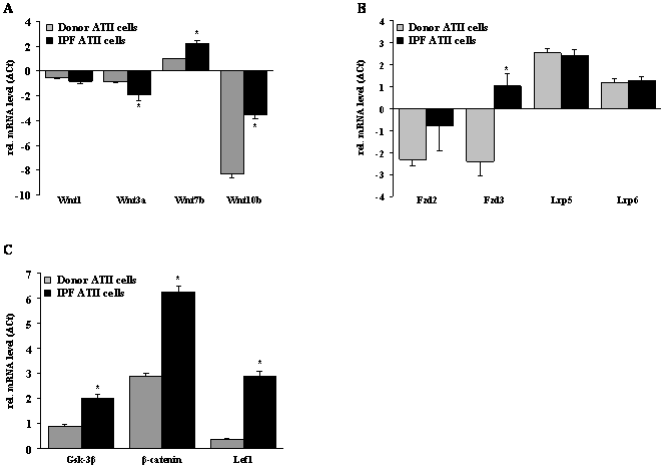
The mRNA expression profile of canonical Wnt signaling components in primary human ATII cells. Primary human ATII cells were isolated from lung tissues of donor and IPF patients as described in Material and Methods. The mRNA levels of Wnt1, 3a, 7b, and 10b (a), the receptors Fzd2 and 3, and Lrp5 and 6 (b), and Gsk-3β, β-catenin, and Lef1 (c) in ATII cells were assessed by qRT-PCR. Results are derived from 3 different cell isolations each and presented as mean±s.e.m., * p<0.05.

To further corroborate these results, we quantified cell-specific gene expression of Wnt/β-catenin signaling components in primary human ATII cells and fibroblasts derived from IPF patients or transplant donors, using qRT-PCR (Figure 6). As depicted in Figure 6a, we observed increased mRNA expression of Wnt7b and 10b in ATII cells from IPF patients. Wnt3a was downregulated in IPF ATII cells, while Wnt1 levels remained unchanged. Furthermore, we observed significantly increased mRNA levels of the receptor Fzd3 and the intracellular signaling molecules Gsk-3β, β-catenin, and Lef1 in IPF ATII cells (Figure 6b and 6c, respectively). Interestingly, we routinely observed higher expression levels of these components in primary ATII cells compared with fibroblasts (data not shown), thereby confirming our localization analysis. In sum, these data revealed the expression of all required Wnt components in the lung, and significant upregulation thereof in IPF, mainly in the bronchial and alveolar epithelium.

**Figure 7:**
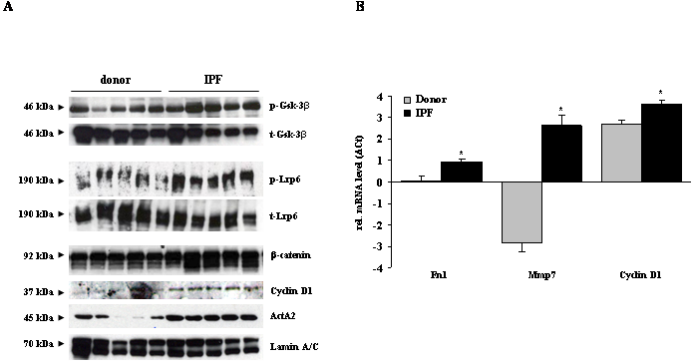
Activity of the canonical Wnt signal pathway in lung homogenates of donor and IPF patients. (a) The expression of active Wnt components in lung homogenates of donor and IPF patients was analyzed by immunoblotting of phosphorylated Gsk-3β and Lrp6, total β-catenin, and the Wnt target gene Cyclin D1. Blotting of total Gsk-3β, Lrp6, and lamin A/C served as loading controls. Immunoblotting of smooth muscle actin (ActA2) was used as a positive control for IPF specimen. (b) The mRNA levels of Fn 1, Mmp 7, and Cyclin D1 were assessed by qRT-PCR. Results are derived from 6 donors and 6 IPF patients and presented as mean±s.e.m., * p<0.05.

To accurately assess, whether the Wnt/β-catenin signaling pathway was activated in IPF, we performed Western blot analysis of phospho-Gsk-3β, phospho-Lrp6, and β-catenin. As presented in Figure 7a, we observed an increased phosphorylation of both, Gsk-3β and Lrp6, in IPF. This is consistent with increased expression of total β-catenin (Figure 7a), and indicative of activated Wnt/β-catenin signaling. Next, we analyzed the expression of the well-characterized Wnt target genes fibronectin (Fn) 1, matrix metalloproteinase (Mmp) 7, and cyclin D1 in IPF lungs. As depicted in Figure 7b, all of these Wnt target genes were upregulated in IPF lung samples, as compared with transplant donor samples.

**Figure 8:**
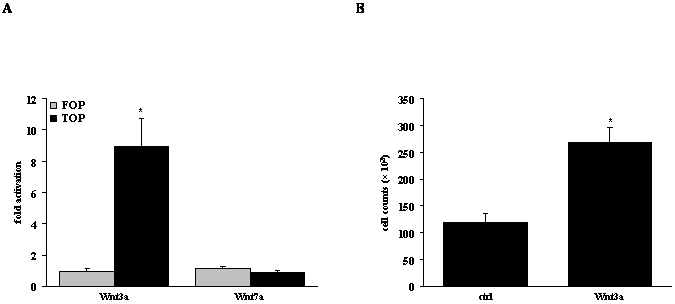
Proliferative effect induced by Wnt3a in alveolar epithelial cells. (a) A549 lung epithelial cell were transiently transfected with FOPflash or TOPflash Wnt reporter constructs (FOP and TOP, respectively), and stimulated with Wnt3a or Wnt7a (at 100 ng/ml each), as indicated. Luciferase expression is plotted as fold activation over unstimulated controls. Results are derived from six independent experiments and presented as mean±s.e.m., * p<0.05. (b) Proliferation of A549 cells was assessed by cell counting 24 h after stimulation with Wnt3a (100 ng/ml). All experiments were performed in quadruplicate, with each condition counted at least three times. Results are presented as mean±s.e.m., * p<0.05.

We then sought to investigate biological effects elicited by Wnt ligands on key cell types involved in IPF pathogenesis. To this end, we assessed proliferation, as well as (myo)fibroblast activation and collagen deposition in A549 lung epithelial cells und NIH-3T3 fibroblasts, respectively. Using a Tcf/Lef-driven reporter gene assay, we first demonstrated that Wnt3a elicited a potent canonical Wnt/β-catenin response, while Wnt7a did not (fold induction of 8.96±1.59 and 0.89±0.09 for Wnt3a and Wnt7a, respectively; Figure 8a). Furthermore, Wnt3a stimulation led to a strong increase of A549 cell proliferation (268×10^3^±28×10^3^ versus 119×10^3^±16×10^3^ for Wnt3a and control, respectively; Figure 8b).

**Figure 9:**
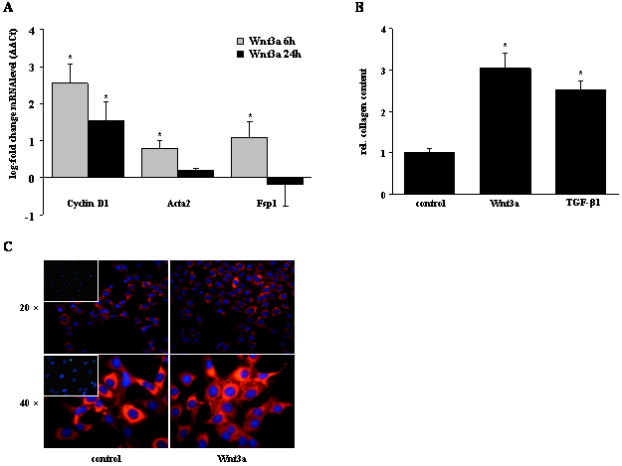
Myofibroblast activation and collagen deposition in response to Wnt3a. (a) The mRNA levels of the Wnt target gene Cyclin D1, or the myofibroblast activation markers smooth muscle actin (Acta2) and fibroblast-specific protein (Fsp) 1 were analyzed by qRT-PCR. Results are derived from 3 independent experiments and presented as mean±s.e.m., * p<0.05. (b) The total collagen content of NIH-3T3 fibroblasts stimulated with Wnt3a (100 ng/ml) or TGF-β1 (2 ng/ml) for 24 h was quantified using the Sircol collagen assay. Results are derived from 5 independent experiments and presented as mean±s.e.m., * p<0.05. (c) Fibroblast collagen expression and localisation after Wnt3a stimulation for 24 h was also assessed by immunofluorescent detection of collagen type 1 (red). Nuclei were visualized by DAPI staining (blue). Control negative immunostainings using iso-type matched IgG instead of a specific primary antibody are demontrated in the inlets of the left panels. Data are representative for at least three independent experiments.

Wnt3a led to a significant induction of the Wnt target gene cyclin D1 and the myofibroblast activation markers smooth muscle actin (Acta2) and fibroblast-specific protein (Fsp) 1, as assessed by qRT-PCR of Wnt3a- or vehicle-treated fibroblasts (Figure 9a). This coincided with increased collagen production, assessed by Sircol assays, in response to Wnt3a, to levels similar to those due to treatment with TGF-β1 (fold induction of 3.07±0.3 and 2.6±0.2 for Wnt3a and TGF-β1, respectively; Figure 9b). This was confirmed by immunofluorescence staining of type I collagen in fibroblasts, demonstrating increased collagen staining in response to Wnt3a (Figure 9c). In contrast, Wnt3a treatment did not affect fibroblast proliferation (data not shown), suggesting that Wnt ligands elicit profibrotic effects in a cell-specific manner on resident lung cells.

## Discussion

IPF is the most common form of the idiopathic interstitial pneumonias. IPF exhibits a poor prognosis and unresponsiveness to currently available therapies, reflecting our limited understanding of the basic mechanisms and mediators implicated in the pathogenesis of this progressive and fatal disease [4], [5]. Historically, inflammatory processes were thought to represent the main trigger of IPF initiation and progression. This view has recently been questioned, due to the ineffectiveness of anti-inflammatory therapies in IPF [10]. Major key pathophysiological events in IPF currently discussed include repetitive alveolar epithelial cell injuries, in the presence or absence of local inflammation, impaired epithelial-mesenchymal crosstalk, and subsequent fibroblast to myofibroblast activation [10], [18]–[20]. These mechanisms are mediated by aberrantly activated signaling molecules that drive the fibrotic process, such as TGF-β, IGF, PDGF, or TNF-α [9], [20].

In this respect, the Wnt signaling system is of particular interest, as it constitutes a developmentally active pathway, which is reactivated in chronic diseases characterized by pathologic tissue remodeling [11]–[13], [21], [22]. Unbiased microarray screens have recently revealed the overexpression of Wnt target genes, including Wnt-induced signaling pathway protein (Wisp) 1, matrix metalloproteinase (Mmp) 7, or secreted frizzled-related protein (Sfrp) 2, in IPF lungs [16], [17], [22]. Furthermore, a recent study localized β-catenin staining to the nuclei of ATII cells and interstitial fibroblasts in IPF lungs [14], suggestive of activated Wnt signaling [23]. Thus, we hypothesized in our study that canonical Wnt signaling is reactivated in IPF lungs, in particular in hyperplastic ATII cells, thus contributing to disease development and progression in IPF. While this has not yet been adressed in the adult lung, canonical Wnt/β-catenin signaling is known to play an essential role in lung development, as lung epithelial cell-specific deletion of β-catenin prevents formation of the distal, but not the proximal airways [24]. Furthermore, epithelial-cell specific expression of constitutively active β-catenin leads to epithelial cell dysplasia and abnormal epithelial differentiation in mice [25].

To this end, we performed a comprehensive analysis of canonical Wnt signaling components at the mRNA and protein level, assessing expression, localization, as well as activity of Wnt ligands, receptors, and intracellular signaling molecules in IPF. We demonstrate that all essential components were expressed in the human lung, and particularly localized to the alveolar and bronchial epithelium in normal, as well as IPF lungs, as demonstrated by qRT-PCR and immunohistochemistry. Lung homogenate, as well as cell-specific analysis of Wnt ligands and receptors demonstrated increased expression of Wnt ligands (compare Figures 1 and 6), with the exception of Wnt1. While Wnt1 was increased in IPF lung homogenates, it was not regulated in IPF ATII cells, suggesting that other cell types, including bronchial epithelial cells or endothelial cells (Figure 2), may serve as the primary source for Wnt1 expression in IPF.

Similar to the Wnt ligands, Gsk-3β and β-catenin were highly expressed in hyperplastic ATII cells and bronchiolar epithelial cells at sites of bronchiolization in IPF. This suggests that Wnt signaling, which is known to drive epithelial cell hyperplasia in non-pulmonary epithelia [13], may regulate ATII cell hyperplasia and increased bronchial epithelial cell proliferation in IPF. Together with our observation that Wnt ligands are mainly secreted by lung epithelial cells, this indicates that Wnt signaling in the adult lung initiates from the epithelium and acts in an autocrine and paracrine fashion on epithelial and mesenchymal cells, respectively. While this has been underappreciated in the normal and diseased adult lung, it represents a very well-documented pathway active in the developing lung [23].

Our current understanding about the functional relevance of Wnt signaling in lung epithelium is largely derived from transgenic animal models. If deleted by homologous recombination in mice, Wnt5a deficiency leads to overexpansion of distal airways and inhibition of lung maturation, accompanied by enlarged intersaccular interstitial compartments [26]. Similarly, the loss of Wnt7b causes perinatal death due to respiratory failure, subsequent to impaired mesenchymal growth and vascular development that is due to defective autocrine and paracrine Wnt signaling by the airway epithelium [27]. Here, we also report that Wnt ligands induce lung epithelial cell proliferation and fibroblast activation and collagen synthesis (Figures 8, 9). These observations further support the importance of properly regulated Wnt signaling for normal epithelial-mesenchymal interactions, and emphasize the impact of dysregulated Wnt signaling in diseases characterized by an impairment thereof, such as IPF.

In this study, we used recombinant Wnt3a to assess the functional effects of canonical Wnt signaling in epithelial cells and fibroblasts. While we observed decreased expression of Wnt3a in IPF homogenates and ATII cells (Figures 1 and 6), we proceeded with Wnt3a for the following reasons: First, Wnt3a has been repeatedly reported to potently stimulate β-catenin-dependent Wnt signaling in vitro [28], and has been recognized as the prototypic Wnt ligand for in vitro stimulations [11]. Second, Wnt3a is one of the few Wnt ligands that is available in active and recombinant form. Other Wnt ligands used in *in vitro* studies, such as Wnt1, are commonly either overexpressed by viral transduction, or supplied in the form of conditioned media harvested from Wnt1 overexpressing cell lines [29], [30]. As we sought to define time-dependent effects of canonical Wnt signaling in our study, we therefore chose to proceed with Wnt3a stimulation.

One of the key questions arising from this study is whether different Wnt isoforms, such as Wnt1 and Wnt3a, are able to induce different effects, or whether they are merely expressed in a distinct spatio-temporal fashion in the lung to elicit different phenotypes, if deleted in mice. In general, a specific biological effect induced by Wnt ligand exposure depends on the expression of distinct Wnt receptors and signaling molecules, distinct ECM molecules such as glypicans, but also on the presence of Wnt inhibitors, such as Dickkopfs, secreted frizzled receptor proteins, or Wnt inhibitory factor [31], [32]. Second, different affinities of Wnt ligands for distinct receptor subtypes have been shown to encode signal specificity [32]–[34]. Third, crosstalk between Wnt/β-catenin signaling with other pathways, such as the TGF-β pathway, may induce tissue- and cell-type-specific effects of relevance to IPF. Of interest, it has recently been demonstrated that the Wnt/β-catenin signaling compounds axin and GSK-3 impact TGF-β signaling via controling Smad3 protein stability [35].

We also demonstrated increased levels of β-catenin in IPF, which were predominantly localized to bronchial epithelial and hyperplastic ATII cells. In contrast to a previous investigation presenting increased nuclear β-catenin staining in bronchial epithelial and ATII cells, as well as interstitial fibroblasts [14], we did not observe widespread nuclear staining of β-catenin using immunohistochemistry in our study. This may be due to different antibody or tissue preparations in these two studies, but moreover suggests that Wnt activation in IPF is more subtle than previously assumed. This may also indicate that only a minority of the cellular β-catenin content in epithelial cells is responsible for Wnt signaling, while the majority of β-catenin molecules is present within the cytosol and at cell-cell contacts [36], [37]. Therefore, to generate a more comprehensive yet concise view of Wnt signaling activity in IPF, we sought to combine expression and localization analysis of Wnt signaling components, paired with phosphorylation analysis of Lrp6 and Gsk-3β, together with quantitative expression analysis of Wnt target genes.

In sum, we report increased functional Wnt signaling in IPF, documented by increased phosphorylation of Lrp6 and Gsk-3β, which has recently been demonstrated to present as a most sensitive indicator of Wnt activity in tissue sections [38], [39]. We also observed increased expression of the Wnt target genes Cyclin D1, Mmp 7, and Fibronectin 1. In particular, Mmp 7 and Fibronectin 1 have recently been assigned a causative role in pulmonary fibrosis and shown to be expressed in interstitial fibroblasts and ATII cells [40], [41]. The Wnt signaling pathway may therefore present as a novel pathogenic system re-activated during chronic tissue remodeling observed in IPF [22]. As pointed out in this study, Wnt ligands are secreted in a cell-specific fashion, but act on a multitude of adjacent cell types, thereby modifying Wnt ligand activity by cellular traps. Future *in vitro* and *in vivo* studies will undoubtedly shed more light on the mechanistic principles underlying Wnt activation in IPF, and whether therapeutic modulation thereof will present as a suitable therapeutic tool in this devastating disease.

## Materials and Methods

### Antibodies and reagents

The following antibodies were used in this study: Total β-catenin (#9562), phospho-S9- and total Gsk-3β (#9336 and #9315, respectively), phospho- and total Lrp6 (#2568 and #2560, respectively; all from Cell Signaling Technology, Beverly, MA), Wnt1 (ab15251, Abcam, Cambridge, UK), Wnt3a (38-2700, Zymed Laboratories/Invitrogen, Carlsbad, CA), CyclinD1 (06-137, Upstate, Temecula, CA), α-smooth muscle actin (SMA, A2547, Sigma-Aldrich, Saint Louis, MO), collagen type 1 (T40777R, Biodesign, Saco, ME) and Lamin A/C (sc-20681, Santa Cruz Biotechnology, Santa Cruz, CA). Dulbecco's modified Eagle's medium (DMEM) and fetal calf serum (FCS) were obtained from Invitrogen. Recombinant Wnt3a and Wnt7a was purchased from R&D Systems.

### Human tissues

Lung tissue biopsies were obtained from 15 IPF patients with histological usual interstitial pneumonia (UIP) pattern (4 females, 11 males; mean age = 58±8 years; mean VC = 48%±7%; mean TLC = 50%±5%; mean DL_CO_/VA = 23%±3%; O_2_ = 2–4 l/min; Pa_O2_ = 49–71 mmHg, Pa_CO2_ = 33–65 mmHg) and 9 control subjects (organ donors; 4 females, 5 males; mean age 42±10 years). Individual patient characteristics are shown in Table 1. Samples were immediately snap frozen or placed in 4% (w/v) paraformaldehyde after explantation. The study protocol was approved by the Ethics Committee of the Justus-Liebig-University School of Medicine (AZ 31/93). Informed consent was obtained in written form from each subject for the study protocol.

**Table 1:** Characteristics of IPF patients with UIP pattern.

No.	Diagnosis	Gender	Age (yr)	VC (%)	DL_CO_/VA (%)	TLC (%)	O_2_ (l/min)	Pa_O2_ (mmHg)	Pa_CO2_(mmHg)
1	IPF (UIP)	male	63	56%	33%	48%	3	52	33
2	IPF (UIP)	male	62	50%	26%	52%	3	49	38
3	IPF (UIP)	male	58	49%	na	na	na	na	na
4	IPF (UIP)	male	65	59%	20%	42%	3	53	38
5	IPF (UIP)	male	65	59%	20%	42%	4	69	41
6	IPF (UIP)	male	43	48%	27%	51%	na	na	na
7	IPF (UIP)	male	71	40%	24%	46%	na	na	na
8	IPF (UIP)	male	64	59%	22%	52%	2	58	38
9	IPF (UIP)	male	60	51%	18%	49%	2	59	39
10	IPF (UIP)	male	65	51%	20%	66%	2	53	38
11	IPF (UIP)	male	44	47%	25%	55%	2	36	35
12	IPF (UIP)	female	43	40%	na	na	2	54	35
13	IPF (UIP)	female	42	50%	17%	58%	3	52	36
14	IPF (UIP)	female	66	29%	23%	45%	4	56	45
15	IPF (UIP)	female	62	27%	na	48%	4	71	65

VC = vital capacity, TLC = total lung capacity, DL_CO_/VA = diffusing capacity of the lung for CO per unit of alveolar volume (all in % predicted), Pa_O2/CO2_ = partial pressure of O_2_/CO_2_ in the arterial blood.

### Cell culture

Human alveolar epithelial type II (ATII) cells were isolated, as previously described [42]. The purity and viability of ATII cell preparations was consistently >90% and >95%, respectively. Primary human fibroblasts were generated by explant cultures as previously described. Identification of fibroblasts was based on the expression of vimentin, collagen, and αSMA [43]. The NIH-3T3 murine fibroblast cell line [German Collection of Microorganisms and Cell Cultures (DSMZ, Braunschweig, Germany)] and the human lung epithelial cell line A549 (ATCC #CCL-185) were maintained in DMEM containing 10% FCS and cultured in a humidified atmosphere of 5% CO_2_ at 37°C.

### Reverse transcription and quantitative real-time PCR

Total RNA was extracted using Qiagen extraction kits according to the manufacturer's protocol, and cDNAs were generated by reverse transcription using SuperScript™ II (Invitrogen) [43], [44]. Quantitative (q)RT-PCR was performed using fluorogenic SYBR Green and the Sequence Detection System Fast 7500 (PE Applied Biosystems), as previously described. *Hprt1* and *Pbgd*, ubiquitously and equally expressed genes free of pseudogenes, were used as a reference gene in all human and mouse qRT-PCR reactions, respectively. PCR was performed using the primers listed in Table 2, at a final concentration of 200 nM. Relative transcript abundance of a gene is expressed in ΔCt values (ΔCt = Ct^reference^ – Ct^target^). Relative changes in transcript levels compared to controls are ΔΔCt values (ΔΔCt = ΔCt^treated^ – ΔCt^control^). All ΔΔCt values correspond approximately to the binary logarithm of the fold change as mentioned in the text. When relative transcript abundance is of information, expression levels are given in ΔCt levels.

**Table 2:** Primer sequences and amplicon sizes.

Gene	Accession		Sequences (5′→3′)	Length	Amplicon
β-Catenin	NM001904	for	AAGTGGGTGGTATAGAGGCTCTTG	24 bp	77 bp
		rev	GATGGCAGGCTCAGTGATGTC	21 bp	
Cyclin D1	NM053056	for	CCGAGAAGCTGTGCATCTACAC	22 bp	94 bp
		rev	AGGTTCCACTTGAGCTTGTTCAC	23 bp	
Fn1	NM212482	for	CCGACCAGAAGTTTGGGTTCT	21 bp	81 bp
		rev	CAATGCGGTACATGACCCCT	20 bp	
Fzd1	NM003505	for	AGCGCCGTGGAGTTCGT	17 bp	64 bp
		rev	CGAAAGAGAGTTGTCTAGTGAGGAAAC	27 bp	
Fzd2	NM001466	for	CACGCCGCGCATGTC	15 bp	63 bp
		rev	ACGATGAGCGTCATGAGGTATTT	23 bp	
Fzd3	NM017412	for	GGTGTTCCTTGGCCTGAAGA	20 bp	72 bp
		rev	CACAAGTCGAGGATATGGCTCAT	23 bp	
Fzd4	NM012193	for	GACAACTTTCACACCGCTCATC	22 bp	164 bp
		rev	CCTTCAGGACGGGTTCACA	19 bp	
Hprt1	NM000194	for	AAGGACCCCACGAAGTGTTG	20 bp	137 bp
		rev	GGCTTTGTATTTTGCTTTTCCA	22 bp	
Gsk-3β	NM002093	for	CTCATGCTCGGATTCAAGCA	20 bp	86 bp
		rev	GGTCTGTCCACGGTCTCCAGTA	22 bp	
Lef1	NM016269	for	CATCAGGTACAGGTCCAAGAATGA	24 bp	93 bp
		rev	GTCGCTGCCTTGGCTTTG	18 bp	
Lrp5	NM002335	for	GACCCAGCCCTTTGTTTTGAC	21 bp	138 bp
		rev	TGTGGACGTTGATGGTATTGGT	22 bp	
Lrp6	NM002336	for	GATTCAGATCTCCGGCGAATT	21 bp	83 bp
		rev	GGCTGCAAGATATTGGAGTCTTCT	24 bp	
Mmp7	NM002423	for	GAACGCTGGACGGATGGTA	19 bp	64 bp
		rev	CAAGTTCATGAGTTGCAGCATACA	24 bp	
Tcf3	NM031283	for	ACCATCTCCAGCACACTTGTCTAATA	26 bp	71 bp
		rev	GAGTCAGCGGATGCATGTGA	20 bp	
Tcf4	NM030756	for	GCGCGGGATAACTATGGAAAG	21 bp	89 bp
		rev	GGATTTAGGAAACATTCGCTGTGT	24 bp	
Wnt1	NM005430	for	CTCATGAACCTTCACAACAACGA	23 bp	80 bp
		rev	ATCCCGTGGCACTTGCA	17 bp	
Wnt2	NM003391	for	CCTGATGAATCTTCACAACAACAGA	25 bp	78 bp
		rev	CCGTGGCACTTGCACTCTT	19 bp	
Wnt3a	NM033131	for	GCCCCACTCGGATACTTCTTACT	23 bp	98 bp
		rev	GAGGAATACTGTGGCCCAACA	21 bp	
Wnt7b	NM058238	for	GCAAGTGGATTTTCTACGTGTTTCT	25 bp	65 bp
		rev	TGACAGTGCTCCGAGCTTCA	20 bp	
Wnt10b	NM003394	for	GCGCCAGGTGGTAACTGAA	19 bp	59 bp
		rev	TGCCTGATGTGCCATGACA	19 bp	
mCyclinD1	NM007631	for	ATGCCAGAGGCGGATGAGA	19 bp	98 bp
		rev	ATGGAGGGTGGGTTGGAAAT	20 bp	
mFsp1	NM011311	for	AGGAGCTACTGACCAGGGAGCT	22 bp	102 bp
		rev	TCATTGTCCCTGTTGCTGTCC	20 bp	
mActa2	NM007392	for	GCTGGTGATGATGCTCCCA	19 bp	80 bp
		rev	GCCCATTCCAACCATTACTCC	21 bp	
mPbgd	NM007392	for	ATGTCCGGTAACGGCGGC	18 bp	139 bp
		rev	GGTACAAGGCTTTCAGCATCGC	22 bp	

All primer sets worked under identical real-time PCR cycling conditions with similar efficiencies to obtain simultaneous amplification in the same run. Sequences were taken from GeneBank, all accession numbers are denoted.

### Immunohistochemistry

Human lungs were placed in 4% (w/v) paraformaldehyde after explantation, and processed for paraffin embedding. Sections (3 µm) were cut, mounted on slides, subjected to antigen retrieval, and quenching of endogenous peroxidase activity using 3% (v/v) H_2_O_2_ for 20 min. Immune complexes were visualized using suitable peroxidase-coupled secondary antibodies, according to the manufacturer's protocol (Histostain *Plus* Kit; Zymed/Invitrogen) [43], [44].

### Immunofluorescence

NIH-3T3 cells were plated on chamber slides, fixed with acetone/methanol (1∶1), and blocked for unspecific binding sites with 3% (m/vol) BSA. Fixed cells were incubated with the indicated primary antibodies for 60 min in PBS containing 0.1% (m/vol) BSA. Indirect immunofluorescence was performed by incubation with Alexa 555-conjugated secondary antibodies (Molecular Probes, Eugene, Oregon). Nuclei were visualized by 4,6-diamidino-2-phenylindole staining (DAPI; Roche Diagnostics).

### Western blot analysis

Human lung tissue specimens were homogenized in extraction buffer [20 mM Tris-Cl, 150 mM NaCl, 1 mM EDTA, 1 mM EGTA, 1% (v/v) Triton X-100, supplemented with Complete™ Proteinase Inhibitor Cocktail (Merck Biosciences)] and whole proteins were extracted by centrifugation (12.000× g) for 10 min at 4°C, as described previously [43]. Samples containing 25 µg of protein were separated by electrophoresis on a 10% SDS-polyacrylamide gels. The separated proteins were transferred to nitrocellulose membranes (Invitrogen), blocked with 5% skim milk, and incubated with the indicated antibodies. Proteins were then visualized by enhanced chemiluminescence detection (ECL, Amersham Biosciences, Uppsala, Sweden), as reported. Prior to reprobing, nitrocellulose membranes were incubated with stripping buffer [100 mM 2-mercaptoethanol, 2% SDS, and 62.5 mM Tris-HCl (pH 6.7)] at 50 °C for 30 min.

### Reporter gene assay

Lung epithelial A549 cells were transiently transfected with the reporter construct TOPflash or FOPflash (kindly provided by R. Moon, University of Washington, Seattle) using Lipofectamine™ 2000 (Invitrogen). The TOPflash construct contains eight Tcf/Lef binding sites upstream of a minimal TA viral promoter and the firefly luciferase cDNA. The FOPflash construct is identical with the exception that it contains mutated copies of TCf/Lef binding sites and is used as a control for measuring nonspecific activation of the reporter construct. Treatment with Wnt3a (100 ng/ml) or Wnt7a (100 ng/ml) for 24 h was performed 4 h after transfection. Luciferase activities were determined using the Dual Luciferase Assay System (Promega) on a Fusion™ luminometer (Packard BioScience).

### Collagen assay

NIH-3T3 fibroblasts were plated at a density of 30.000 cells/well in 6-well plates, synchronized for 24 h in serum-free medium, and treated for 24 h as indicated. Total collagen content was determined using the Sircol Collagen Assay kit (Biocolor, Belfast, Northern Ireland). Equal amounts of protein lysates were added to 1 ml of Sircol dye reagent, followed by 30 min of mixing. After centrifugation at 10.000× g for 10 min, the supernatant was carefully aspirated and 1 ml of Alkali reagent was added. Samples and collagen standards were then read at 540 nm on a spectrophotometer (Bio-Rad). Collagen concentrations were calculated using a standard curve generated by using acid-soluble type 1 collagen.

### Statistical analysis

All ΔCt values obtained from real-time RT-PCR were analyzed for normal distribution using the Shapiro-Wilk test, using assignment of a normal distribution with p>0.05. Normality of data was confirmed using quantile-quantile plots. The means of indicated groups were compared using two-tailed Student's *t*-test, or a one-way analysis of variance (ANOVA) with Tukey HSD post hoc test for studies with more than 2 groups. All p values obtained from multiple tests were adjusted using the procedure from Hochberg and Benjamini [45]. Results were considered statistically significant when p<0.05.
